# NGX-4010, a capsaicin 8% patch, for the treatment of painful HIV-associated distal sensory polyneuropathy: integrated analysis of two phase III, randomized, controlled trials

**DOI:** 10.1186/1742-6405-10-5

**Published:** 2013-01-28

**Authors:** Stephen Brown, David M Simpson, Graeme Moyle, Bruce J Brew, Giovanni Schifitto, Nicholas Larbalestier, Chloe Orkin, Martin Fisher, Geertrui F Vanhove, Jeffrey K Tobias

**Affiliations:** 1 AIDS Research Alliance, Los Angeles, CA, USA; 2 Mount Sinai School of Medicine, New York, NY, USA; 3 Chelsea and Westminster Hospital, London, United Kingdom; 4 St Vincent’s Hospital, Darlinghurst, Australia; 5 University of Rochester, Rochester, NY, USA; 6 St Thomas’ Hospital, London, United Kingdom; 7 Andrews Out Patients Unit, London, United Kingdom; 8 HIV/GUM Research, Elton John Centre, Brighton, United Kingdom; 9 NeurogesX, Inc, San Mateo, CA, USA

**Keywords:** NGX-4010, Capsaicin 8% patch, HIV-associated distal sensory polyneuropathy, Neuropathic pain

## Abstract

**Background:**

HIV-associated distal sensory polyneuropathy (HIV-DSP) is the most frequently reported neurologic complication associated with HIV infection. NGX-4010 is a capsaicin 8% dermal patch with demonstrated efficacy in the treatment of HIV-DSP. Data from two phase III, double-blind studies were integrated to further analyze the efficacy and safety of NGX-4010 and explore the effect of demographic and baseline factors on NGX-4010 treatment in HIV-DSP.

**Methods:**

Data from two similarly designed studies in which patients with HIV-DSP received NGX-4010 or a low-concentration control patch (capsaicin 0.04% w/w) for 30 or 60 minutes were integrated. Efficacy assessments included the mean percent change from baseline in Numeric Pain Rating Scale (NPRS) scores to Weeks 2–12. Safety and tolerability assessments included adverse events (AEs) and pain during and after treatment.

**Results:**

Patients (n = 239) treated with NGX-4010 for 30 minutes demonstrated significantly (p = 0.0026) greater pain relief compared with controls (n = 100); the mean percent change in NPRS scores from baseline to Weeks 2–12 was −27.0% *versus −*15.7%, respectively. Patients who received a 60-minute application of NGX-4010 (n = 243) showed comparable pain reductions (−27.5%) to patients treated for 30 minutes, but this was not statistically superior to controls (n = 115). NGX-4010 was effective regardless of gender, baseline pain score, duration of HIV-DSP, or use of concomitant neuropathic pain medication, although NGX-4010 efficacy was greater in patients not receiving concomitant neuropathic pain medications. NGX-4010 was well tolerated; the most common AEs were application-site pain and erythema, and most AEs were mild to moderate. The transient increase in pain associated with NGX-4010 treatment decreased the day after treatment and returned to baseline by Day 2.

**Conclusions:**

A single 30-minute application of NGX-4010 provides significant pain relief for at least 12 weeks in patients with HIV-DSP and is well tolerated.

**Trial registration:**

C107 = NCT00064623; C119 = NCT00321672

## Background

HIV-associated distal sensory polyneuropathy (HIV-DSP) is the most frequently reported neurologic complication associated with HIV infection. It can be caused by the virus itself [1] or by the use of antiretroviral drugs; for example, some nucleoside reverse transcriptase inhibitors have been shown to have a dose-dependent toxic effect in 15–30% of patients [1]. The clinical presentation of HIV-DSP is similar regardless of whether it is caused by HIV itself or is due to the toxicity of antiretroviral drugs.

HIV-DSP presents with predominantly symmetrical signs of distal sensory loss and reduction or loss of ankle reflexes. Symptomatic HIV-DSP reflects the involvement of both small and large sensory nerve fibers and includes distal painful dysesthesias, allodynia, severe burning pain, pins and needles, and numbness [1,2]. These symptoms usually begin in the feet and may progress bilaterally up the legs and to the arms in severe HIV-DSP.

In a recent US study, 881 of 1,539 (57%) individuals infected with HIV showed evidence of HIV-sensory neuropathy, which encompasses HIV-DSP caused by the virus and toxic DSP due to dideoxynucleoside antiretroviral therapy [3]. Of those with DSP, 38% reported neuropathic pain [4]. Very few neuropathic pain treatments, however, have demonstrated efficacy in clinical trials for patients with HIV-associated neuropathy. For example, the tricyclic antidepressant amitriptyline [5], the anticonvulsant pregabalin [6], and topical treatments such as lidocaine [7] and low-dose (0.075%) capsaicin cream [8] have all failed to show significant pain relief when compared with controls. By contrast, three small, randomized, placebo-controlled studies showed that smoking cannabis reduced daily pain compared with smoking identical placebo cigarettes from which the cannabinoids had been removed [9-11]. Other neuropathic pain therapies such as duloxetine and venlafaxine have not been studied in patients with HIV-DSP [12] while the results of a single published study investigating opioid use among patients with HIV suggested those receiving opioids experienced more pain than those patients who did not receive opioids [13]. Furthermore, many of these agents, which are not approved by the Food and Drug Administration or the European Medicines Agency, are associated with unwanted side effects and burdensome treatment regimens [12]. Sedation and dizziness are common adverse events (AEs) associated with gabapentin and pregabalin, whereas nausea can be common with duloxetine [12]. Gabapentin and pregabalin also require slow dose titration, as does amitriptyline [12]. In addition, cumbersome regimens must be followed for treatment with lidocaine patches and low-dose capsaicin creams; the lidocaine patch is licensed to be worn for 12 hours followed by a 12-hour treatment break [12], and capsaicin cream requires application several times a day [8]. A recent systematic review of randomized controlled studies concluded that evidence of efficacy exists only for capsaicin 8%, smoked cannabis, and recombinant-human nerve growth factor (rhNGF) [14]. However, rhNGF did not demonstrate evidence for the expected nerve fiber regeneration [15] and was not developed further for clinical use.

NGX-4010 is a capsaicin 8% (w/w) dermal patch licensed in 2009 in the EU for the treatment of peripheral neuropathic pain in non-diabetic adults either alone or in combination with other medicinal products for pain; and indicated in the US for the management of neuropathic pain associated with post-herpetic neuralgia. NGX-4010 was developed to rapidly deliver a high dose of capsaicin directly to the source of pain with a single application. Capsaicin is a highly selective agonist for the Transient Receptor Potential Vanilloid 1 (TRPV1) receptor, which is a ligand-gated non-selective cation channel that is highly expressed in nociceptors and is critical for pain transmission and modulation [16]. As a component of small nerve fibers, C fibers are involved in HIV-DSP [17] and, as TRPV1 expression is altered on C fibers following nerve injury [18], these fibers may play a role in the neuropathic pain experienced by individuals with HIV-DSP. TRPV1 is therefore a logical target for treating neuropathic pain. Exposure of TRPV1 receptors to high concentrations of capsaicin initially causes depolarization, action potential initiation, and burning pain [19,20]. This is followed by a defunctionalization and reduction in the density of epidermal nerve fibers, resulting in inhibition of pain transmission [19-21]. The effect is reversible, with regrowth of epidermal nerve fibers evident 12 weeks after exposure to capsaicin [21].

Two similarly designed phase III, double-blind, controlled studies have investigated the efficacy of NGX-4010 in patients with HIV-DSP. These studies demonstrated that treatment with NGX-4010 was well tolerated and resulted in a reduction in pain over 12 weeks [22,23] (Table 1). Study C107 investigated the efficacy of NGX-4010 applied for 30, 60, or 90 minutes and demonstrated that the 30-minute NGX-4010 application resulted in a statistically significant reduction in pain; there was no evidence of increased efficacy with longer application durations [22]. Study C119 investigated the efficacy of NGX-4010 applied for 30 or 60 minutes and demonstrated a greater pain reduction in the 30-minute NGX-4010 group *versus* the 30-minute control group. Although this did not attain statistical significance using a prespecified analysis of covariance (ANCOVA) model, it did show a significant difference (p = 0.035) when a post hoc non-parametric test was used [23]. In neither study did the 60-minute treatment group show a significant difference as compared with control.

**Table 1:** **Summary of randomized**, **double**-**blind**, **controlled trials of NGX**-**4010 in patients with HIV**-**DSP**

**Trial**	**Number of patients**		**Dose**	**Mean percent change in NPRS “average pain for past 24 hours” during Weeks 2**–**12 post application compared with baseline (%)**	
	**NGX**-**4010**	Control patch^*^			
C107 [22]	225	82	Single 30-, 60-, or 90-minute application of NGX-4010 patch *versus* low-concentration (0.04%) capsaicin control patch	30 minutes	−27.7
					(p = 0.0007^†^*versus* control)
				60 minutes	−15.8
					(p = 0.291^†^*versus* control)
				90 minutes	−24.7
					(p = 0.0046^†^*versus* control)
				Pooled	−22.8
					(p = 0.0026^†^*versus* control)
C119 [23]	332	162	Single 30- or 60-minute application of NGX-4010 patch *versus* low-concentration (0.04%) capsaicin control patch	30 minutes	−26.2
					(p = 0.1031^‡^*versus* control)
				60 minutes	−32.8
					(p = 0.4884^‡^*versus* control)
				Pooled	−29.5
					(p = 0.0967^‡^*versus* control)

*NPRS* Numeric Pain Rating Score.

^*^A low-concentration capsaicin patch (0.04% w/w) was used as a control to ensure effective blinding in the randomized studies.

^†^p-value was computed using gender-stratified ANCOVA to test for a difference between the NGX-4010 group and the total control group, with baseline pain score, pre-topical anesthetic pain score, and percent change in pain score after topical anesthetic treatment as covariates.

^‡^p-value was computed using a gender-stratified ANCOVA to compare differences between each NGX-4010 group and the respective control group, with baseline pain as the covariate.

To analyze in more detail the efficacy and safety of NGX-4010 in patients with HIV-DSP, an integrated analysis of the 30- and 60-minute NGX-4010 applications from the two double-blind, controlled HIV-DSP studies was performed. This integrated analysis also investigated the effects of demographic factors, disease duration and severity, and the use of concomitant neuropathic pain medication, which are often difficult to evaluate in individual studies due to the limited sample size.

## Results

### Patients

**Figure 1:**
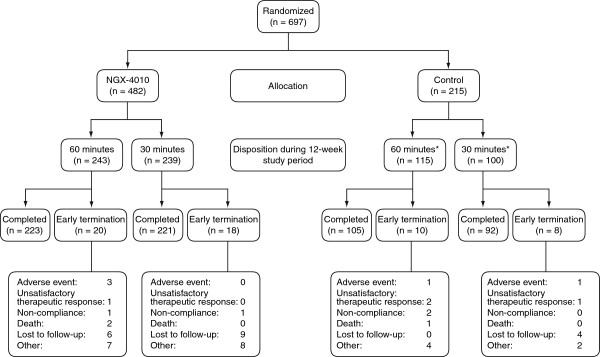
**Disposition of all patients from both phase III HIV**-**DSP studies used in the integrated analysis.** *One patient randomly assigned to receive the 30-minute control treatment received the 60-minute control treatment.

The integrated analysis comprised data from 482 and 215 patients treated with NGX-4010 and a control patch, respectively (Figure 1). Of the patients treated with NGX-4010, 239 and 243 received a 30- and 60-minute application, respectively. Among the control patients, 100 and 115 received a 30- and 60-minute application, respectively. One patient, in the control group, was randomly assigned to receive a 30-minute treatment, but actually received a 60-minute treatment.

Gender, age, race, baseline Numeric Pain Rating Scale (NPRS) score, CD4 cell count, viral load, and the percentage of patients receiving some form of concomitant neuropathic pain treatment at baseline were similar between the two phase III studies and between treatment groups within the two studies (NeurogesX unpublished data) [22,23]. The average duration of HIV-DSP was slightly longer in study C119 (means ranged from 5.7– 6.6 years [23]) compared with study C107 (means ranged from 4.2–5.4 years [22]). Patient demographics and baseline characteristics for the integrated dataset were similar between the NGX-4010 and control groups (Table 2).

**Table 2:** **Demographic and baseline characteristics from the integrated population of the two phase III HIV**-**DSP studies**

		**NGX-4010**	**NGX-4010**		**Control**	**Control**
	**NGX-4010 total**	**30 minutes**	**60 minutes**	**Control total**	**30 minutes**	**60 minutes**
	**(n = 482)**	**(n = 239)**	**(n = 243)**	**(n = 215)**	**(n = 100)**	**(n = 115)**
Age, mean (SD), years	49 (8)	50 (9)	49 (8)	50 (8)	49 (8)	50 (9)
Male, n (%)	426 (88)	205 (86)	221 (91)	193 (90)	90 (90)	103 (90)
White, n (%)	315 (65)	156 (65)	159 (65)	144 (67)	68 (68)	76 (66)
Duration of pain, mean (SD), years	5.8 (3.9)	5.6 (3.7)	6.0 (4.1)	5.6 (4.1)	5.9 (3.9)	5.4 (4.4)
Baseline NPRS score, mean (SD)	6.0 (1.6)	6.0 (1.6)	6.0 (1.5)	5.9 (1.5)	6.0 (1.5)	5.8 (1.5)
Receiving concomitant neuropathic pain medication at study entry,^*^ n (%)	339 (70)	180 (75)	159 (65)	143 (67)	72 (72)	71 (62)
Receiving Ntox antiretroviral therapy at baseline,^†^ n (%)	52 (11)	25 (10)	27 (11)	18 (8)	8 (8)	10 (9)
CD4 count, mean x10^6^/l (SD)	419 (242)	437 (235)	402 (247)	468 (314)	479 (291)	458 (334)
Viral load, HIV RNA copies/ml, median	400	400	400	400	400	400
(range)	(40–5,510,520)	(40–610,677)	(40–5,510,520)	(40–664,583)	(40–664,583)	(40–460,000)

*NPRS* Numeric Pain Rating Scale, *SD* standard deviation.

^*^A patient was defined as receiving concomitant neuropathic pain medication if he or she was taking an anticonvulsant, non-SSRI antidepressant, or opioid on Day −1 and for a total duration of at least 7 consecutive days.

^†^A patient was defined as receiving antiretroviral therapy if he or she was taking neurotoxic antiretroviral medications for at least 8 weeks prior to the screening date.

The percentage of patients terminating the studies before the end of the 12-week double-blind period was 8% for the combined NGX-4010 group and 8% for the combined control group (Figure 1). Reasons for premature termination were AEs, being lost to follow-up, death, non-compliance, unsatisfactory therapeutic response, and other. Five patients (three in the 60-minute NGX-4010 group, one in the 60-minute control group, and one in the 30-minute control group) withdrew from the study due to AEs. In only two of these patients were the AEs judged to be related to study medication and both consisted of application-site pain. Both patients were in the 60-minute NGX-4010 group. Three patients died during the 12-week observation period; two deaths in the 60-minute NGX-4010 group were due to sepsis and pre-existing arteriosclerosis and one death in the 60-minute control group was due to a presumed drug overdose. No deaths were considered to be related to study drug treatment.

### Efficacy

Data for the primary endpoint, the mean change in NPRS score from baseline to Week 2–12, for the integrated 30- and 60-minute treatment groups, along with those of other efficacy endpoints are shown in Table 3. These analyses demonstrated that the 30- and 60-minute NGX-4010 doses provided comparable pain reduction (−26.9% and −27.9%, respectively). Only the 30-minute dose was statistically superior to the low-concentration capsaicin control; the 60-minute NGX-4010 group showed a similar pain reduction to the 60-minute control group.

**Table 3:** **Integrated efficacy data for the 30**- **and 60**-**minute treatment groups**

	**NGX-4010**			**Control**		
	**Total**	**30 minutes**	**60 minutes**	**Total**	**30 minutes**	**60 minutes**
	**(n = 482)**	**(n = 239)**	**(n = 243)**	**(n = 215)**	**(n = 100)**	**(n = 115)**
LS mean change (SE) in NPRS score from baseline to Weeks 2–12	−27.4 (1.4)	−26.9 (2.1)	−27.9 (2.0)	−20.0 (2.1)	−15.8 (3.0)	−24.2 (2.9)
95% CI of LS mean	−30.1, –24.7	−30.8, –23.0	−31.7, –24.0	−24.1, –15.9	−21.8, –9.8	−29.8, –18.6
p-value^*^	0.0034	0.0024	0.2935	—	—	—
Patients with ≥30% reduction in NPRS score from baseline to Weeks 2–12, n (%)	193 (40)	95 (40)	98 (40)	66 (31)	23 (23)	43 (37)
OR	1.65	2.21	1.22	—	—	—
95% CI of OR	1.15, 2.35	1.29, 3.79	0.77, 1.95	—	—	—
p-value^†^	0.0062	0.0040	0.3949	—	—	—
PGIC at Week 12	n = 438	n = 220	n = 218	n = 196	n = 92	n = 104
Very much/much/slightly improved, n (%)	294 (67)	143 (65)	151 (69)	97 (49)	38 (41)	59 (57)
p-value^‡^	<0.0001	0.0001	0.0333	—	—	—

*CI* confidence interval, *LS* least square, *NPRS* Numeric Pain Rating Scale, *OR* odds ratio, *PGIC* Patient Global Impression of Change, *SE* standard error.

^*^p-value is computed using weighted gender-stratified ANCOVA to test for difference between each NGX-4010 group and the respective control group, with baseline pain, pre-topical anesthetic pain, and percent change in pain during topical anesthetic application as covariates.

^†^p-value is computed using logistic regression to test for difference between each NGX-4010 group and the respective control group, with gender, baseline pain, pre-topical anesthetic pain, and percent change in pain as covariates.

^‡^p-values were computed using Fisher’s exact test comparing each NGX-4010 group and the respective control group.

Similarly, a comparable percentage of patients in the 30- and 60-minute groups (40% for each) responded to NGX-4010 treatment (≥30% decrease in NPRS score from baseline to Weeks 2–12), but only the 30-minute NGX-4010 treatment resulted in a significantly greater percentage of responders compared with controls (Table 3). Patients treated with NGX-4010 for 30 minutes had a 2.2-fold higher likelihood of being a responder compared with patients treated with control for 30 minutes. The reduction in pain in response to NGX-4010 treatment was confirmed by the results of the Patient Global Impression of Change (PGIC). A comparable percentage of patients in the 30- and 60-minute groups (65% and 69%, respectively) felt slightly, much, or very much improved, and this was significantly different from control in both treatment groups (Table 3).

**Figure 2:**
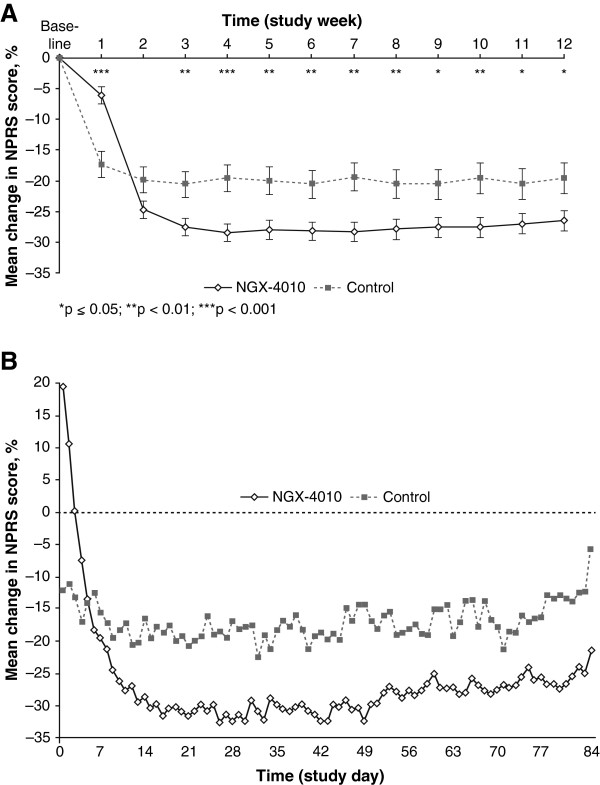
**Pain reduction following a single 30**-**minute application of NGX**-**4010.** (**A**) Weekly analysis of least squares mean change (± standard error) in Numeric Pain Rating Scale (NPRS) score from baseline for the integrated 30-minute treatment group. (**B**) Daily analysis of mean change in NPRS score from baseline for the integrated 30-minute treatment group.

A weekly analysis of change in NPRS score from baseline for the 30-minute treatment groups demonstrated a significantly greater pain reduction for patients treated with NGX-4010 compared with control by Week 2, and this was maintained up to Week 12 (Figure 2A). Figure 2B shows daily mean changes in NPRS score from baseline for the 30-minute treatment groups in more detail. On average, NGX-4010 treatment is associated with a transient increase in pain on Day 0 followed by declining pain scores the following day and a return to baseline by Day 2. After Day 2, patients treated with NGX-4010 experienced a progressive reduction in pain which was greater than that of patients in the control group from Day 5 through to Day 84 (Figure 2B).

**Figure 3:**
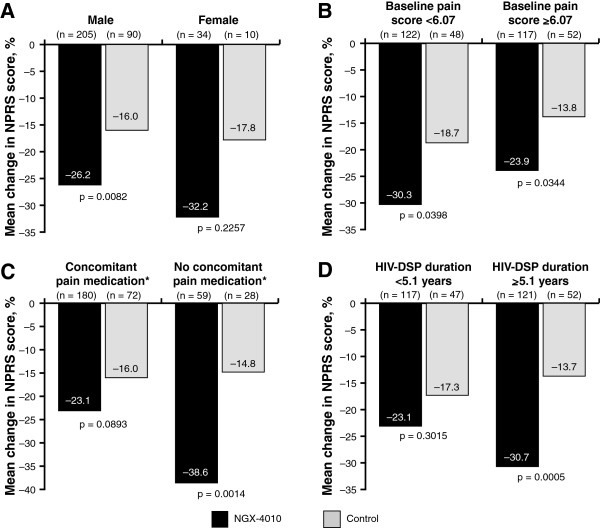
**Mean percent change in NPRS score.** Change in NPRS score from baseline to Weeks 2–12 for the 30-minute treatment groups. Analysis by subgroup: (**A**) gender; (**B**) baseline pain score; (**C**) concomitant neuropathic pain medication use; and (**D**) duration of HIV-DSP. *A patient was defined as receiving concomitant neuropathic pain medication if he or she was taking an anticonvulsant, non-SSRI antidepressant, or opioid on Day −1 and for a total duration of at least 7 consecutive days.

**Figure 4:**
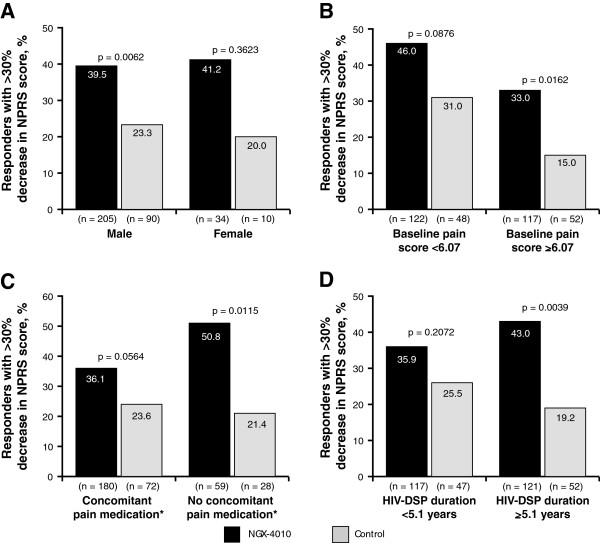
**Percentage of patients with** ≥**30% reduction in NPRS score from baseline to weeks 2–12.** Patients in the 30-minute treatment groups; analysis by subgroup: (**A**) gender; (**B**) baseline pain score; (**C**) concomitant neuropathic pain medication use; and (**D**) duration of HIV-DSP. *Concomitant medication was defined as in Figure 3.

Patients treated with NGX-4010 showed a greater improvement in NPRS scores compared with those treated with control in all subgroups regardless of gender, baseline pain score, concomitant neuropathic pain medication use, and HIV-DSP duration (Figure 3 and 4). Treatment differences between NGX-4010 and control were similar regardless of gender (Figure 3A) or baseline pain score (Figure 3B). Treatment differences between NGX-4010 and control were larger in patients not using concomitant neuropathic pain medications compared with those using concomitant neuropathic pain medications (−23.7% [95% CI: –38.2, –9.3] *versus −*7.0%, [95% CI: –15.1 to 1.1] respectively; Figure 3C). Notably, treatment differences between NGX-4010 and control were larger in patients with a longer duration of HIV-DSP. This was largely due to a greater pain reduction in patients with longer HIV-DSP duration receiving NGX-4010 than those with a shorter duration of HIV-DSP receiving NGX-4010 (Figure 3D).

### Safety

More patients receiving NGX-4010 than control for the combined 30- and 60-minute groups experienced a treatment-related AE (77% *versus* 52%, respectively). The most common (≥2%) treatment-related AEs were application-site reactions, including pain, and erythema (Table 4), which generally appeared to increase with increasing treatment duration. The majority of AEs were mild to moderate in severity; 21% of NGX-4010-treated patients reported a severe AE compared with 8% of patients in the control group. The most common severe AE was application-site pain and the incidence of severe application-site pain was higher in the 60-minute NGX-4010 group (18%) than in the 30-minute NGX-4010 group (10%).

**Table 4:** **The most common treatment**-related AEs*

**System organ class preferred term, n(%)**	**NGX-4010**			**Control**		
	**Total**	**30 minutes**	**60 minutes**	**Total**	**30 minutes**	**60 minutes**
	**(n = 482)**	**(n = 239)**	**(n = 243)**	**(n = 215)**	**(n = 99)**	**(n = 116)**
Number of patients reporting ≥1 treatment-related AE	373 (77)	175 (73)	198 (81)	111 (52)	51 (52)	60 (52)
General disorders and administration-site conditions	369 (77)	173 (72)	196 (81)	108 (50)	51 (52)	57 (49)
Application-site dryness	24 (5)	9 (4)	15 (6)	2 (1)	1 (1)	1 (1)
Application-site erythema	177 (37)	80 (33)	97 (40)	58 (27)	24 (24)	34 (29)
Application-site edema	10 (2)	2 (1)	8 (3)	2 (1)	0	2 (2)
Application-site pain	312 (65)	152 (64)	160 (66)	67 (31)	36 (36)	31 (27)
Application-site papules	20 (4)	9 (4)	11 (5)	1 (<1)	0	1 (1)
Application-site paresthesia	10 (2)	7 (3)	3 (1)	3 (1)	1 (1)	2 (2)
Application-site pruritus	38 (8)	18 (8)	20 (8)	4 (2)	1 (1)	3 (3)
Application-site swelling	17 (4)	4 (2)	13 (5)	4 (2)	2 (2)	2 (2)

*AE* adverse event.

*Includes AEs experienced by ≥2% of patients in any of the total treatment groups.

In the 30-minute treatment groups, 10 (4%) NGX-4010-treated patients and three (3%) control patients reported one or more serious adverse event (SAE). In the 60-minute treatment groups, 19 (8%) NGX-4010-treated patients and eight (7%) control patients reported one or more SAEs. None of the reported SAEs was considered to be related to the study drug.

No clinically important mean changes in hematology, serum chemistry, or vital signs were observed in the NGX-4010 or control groups except for small transient elevations in mean systolic and diastolic blood pressure (<4 mmHg) during and shortly after NGX-4010 application.

### Tolerability

NGX-4010 was well tolerated. Nearly all patients in both the 30- and 60-minute NGX-4010 groups completed at least 90% of the intended NGX-4010 application duration (Table 5). Detailed tolerability assessments demonstrated that mean NPRS scores decreased slightly after application of the topical anesthetic and increased after patch application in all treatment groups. In the 60-minute treatment group, mean NPRS scores increased to pre-topical anesthetic levels by 55 minutes post-patch application and remained at that pain level through 1 hour 55 minutes after patch removal; by contrast, the mean NPRS scores for the 30-minute treatment group remained below pretreatment levels through 1 hour 55 minutes after patch removal (data not shown). Application-site pain appeared to be delayed and, at the end of the treatment day, the mean maximum change in “average pain for the past 24 hours” NPRS score from baseline was 2.2 for those treated with NGX-4010 for 60 minutes and 1.5 for those treated with NGX-4010 for 30 minutes. Increases in NPRS score were transient, with pain reductions observed by Day 3 (Figure 2B). The percentage of patients who were administered medication for treatment-related discomfort on the day of treatment or during the 5 days after treatment was higher in the total NGX-4010 group compared with the control group (71% *versus* 31%, p < 0.0001). The percentage of patients using medication for treatment-related discomfort was lower in the 30-minute NGX-4010 group compared with the 60-minute treatment group (68% and 74%, respectively).

**Table 5:** Summary of tolerability on the treatment day

	**NGX-4010**			**Control**		
	**Total**	**30 minutes**	**60 minutes**	**Total**	**30 minutes**	**60 minutes**
	**(n = 482)**	**(n = 239)**	**(n = 243)**	**(n = 215)**	**(n = 99)**	**(n = 116)**
Change in NPRS score from pre-topical anesthetic time point at the last observation*						
Mean (SD)	1.8 (2.9)	1.5 (3.0)	2.2 (2.8)	−0.2 (2.3)	−0.1 (2.0)	−0.4 (2.5)
p-value^†^	<0.0001	<0.0001	<0.0001	—	—	—
Patients with at least 90% of the intended patch application duration, n (%)	482 (100)	239 (100)	243 (100)	214 (100)	98 (99)	116 (100)
p-value^‡^	0.1340	0.2912	0.2872	—	—	—
Patients using medication for treatment-related discomfort on Days 0–5, n (%)	342 (71)	162 (68)	180 (74)	67 (31)	29 (29)	38 (33)
p-value^§^	<0.0001	<0.0001	<0.0001	—	—	—
Maximum dermal irritation^‖^ score on Day 0, n (%)						
0	198 (41)	111 (46)	87 (36)	121 (56)	56 (57)	65 (56)
1	135 (28)	66 (28)	69 (28)	69 (32)	33 (33)	36 (31)
2	133 (28)	58 (24)	75 (31)	24 (11)	10 (10)	14 (12)
>2	16 (3)	4 (2)	12 (5)	1 (1)	0 (0)	1 (1)
p-value**	<0.0001	0.001	<0.0001	—	—	—

*NPRS* Numeric Pain Rating Scale, *SD* standard deviation.

*Includes the evening of the treatment day.

^†^ p-value was computed using a Wilcoxon rank-sum test comparing the means between each NGX-4010 group and the pooled control group.

^‡^ p-value was computed using a Pearson’s chi-square test comparing the percentages of patients between each NGX-4010 group and the pooled control group.

^§^ p-value was computed using a Fisher’s exact test comparing the percentages of patients between each NGX-4010 group and the pooled control group.

^‖^Dermal irritation assessed by scoring the irritation from 0–7 where 0=no evidence of irritation, 1=minimal erythema, barely perceptible, 2=definite erythema, readily visible, minimal edema or minimal papular response, 3=erythema and papules, 4=definite edema, 5=erythema, edema, and papules, 6=vesicular eruption, and 7=strong reaction spreading beyond test [24].

** p-value was computed using an exact Cochran–Mantel–Haenszel test comparing the distribution of scores between each NGX-4010 group and the pooled control group.

As expected, on the day of treatment there were significant differences in the distribution of maximum dermal assessment scores between the total NGX-4010 and control groups (p < 0.0001). A total of 60% of NGX-4010-treated patients and 44% of control patients had a maximum dermal assessment score of >0. Dermal irritation was commonly mild in the NGX-4010-treatment groups, with dermal assessment scores ≥2 (definite erythema, readily visible, minimal edema, or minimal papular response) being reported in only a few patients (2% and 5%, for the 30- and 60-minute NGX-4010 groups, respectively).

## Discussion

The similarity of the demographic and baseline characteristics of the patients from the two multicenter, randomized, double-blind, controlled phase III studies (Table 2), together with comparable study designs, enrollment, and assessment criteria allowed data from the two studies to be combined. The integrated analyses from the two phase III studies demonstrated that a single 30-minute application of NGX-4010 provides significant pain relief — as demonstrated by a decrease in NPRS score — for patients with HIV-DSP for at least 12 weeks. This decrease in the NPRS score of patients receiving a 30-minute NGX-4010 application was supported by the analysis of patient-reported outcomes using the PGIC. The PGIC provides a global assessment of patient improvement that is independent of NPRS score collection. This measurement is recommended in chronic pain studies by the Initiative on Methods, Measurement, and Pain Assessment in Clinical Trials [25] and is more sensitive to treatment effects in neuropathic pain than pain intensity measurements [26]. Using the PGIC, 12 weeks after a single application of NGX-4010, 65% of patients felt slightly, much, or very much improved compared with 41% of control patients.

The reduction in NPRS score following the 60-minute application of NGX-4010 was not significantly different from control. This is likely due to the large effects of the control patch observed in study C119 [23]. Interestingly, there was a significant improvement using the PGIC in the 60-minute treatment compared with the control group.

The data presented here show no evidence of increased efficacy with increased NGX-4010 treatment duration and support the selection of the 30-minute dose for treatment of peripheral neuropathic pain in patients with HIV-associated neuropathy. By contrast, for patients with post-herpetic neuralgia who typically experience neuropathic pain on the torso as opposed to the extremities, treatment duration is 60 minutes [27,28].

A transient increase in pain compared with baseline was seen on Day 0 in patients receiving NGX-4010, due to the treatment procedure. However, treatment-related pain was short-lived, with NPRS scores for patients treated with NGX-4010 for 30 minutes returning to baseline by Day 2. Thereafter, NPRS scores in patients receiving a 30-minute NGX-4010 application continued to decline and NGX-4010-treated patients achieved a greater pain reduction than those in the control group by Day 5 and on each subsequent day through Day 84. Weekly NPRS scores demonstrated significantly greater reductions in pain for NGX-4010-treated patients compared with controls each week from Week 2 to study completion (Week 12).

Subgroup analyses showed greater pain reduction in patients treated with NGX-4010 compared with those treated with control in all subgroups regardless of gender, baseline pain score, concomitant neuropathic pain medication, and HIV-DSP duration. However, NGX-4010 was more effective in patients not using concomitant neuropathic pain medications compared with those using concomitant neuropathic pain medications. This result is not unexpected as it has been shown previously that the additional reduction of neuropathic pain obtained with an add-on drug is generally not as large as the benefit obtained with monotherapy [29,30]. This may be because some of the pathophysiologic mechanisms implicated in the generation and maintenance of neuropathic pain overlap or converge to a common pathway, resulting in a less than additive effect even for medications that act via different mechanistic pathways. Furthermore, patients already taking several concomitant neuropathic pain medications may be more treatment resistant or more difficult to treat. It is therefore important to note that a significant treatment effect was seen with NGX-4010 compared with control, despite two-thirds of the patients using concomitant neuropathic pain medication at study entry and during the study (Table 2). Gender, baseline pain score, and the duration of HIV-DSP did not influence NGX-4010 efficacy, indicating that pain relief can be achieved with NGX-4010 treatment even in those patients with high pain levels or long disease duration.

Although NGX-4010 application does cause some treatment-related pain, it is generally mild or moderate and transient. The vast majority of patients were able to tolerate >90% of the intended NGX-4010 application. Most common treatment-related AEs were application-site reactions; systemic effects were limited to small, transient elevations in mean systolic and diastolic blood pressure shortly after NGX-4010 application due to treatment-associated increases in pain.

## Conclusions

With the current lack of effective treatment for pain associated with HIV-DSP, a therapy such as NGX-4010, which can deliver rapid and prolonged pain relief for at least 3 months after a single 30-minute application, may be of great benefit to patients with HIV-DSP. In addition to its efficacy, there are few systemic AEs associated with NGX-4010 [31], unlike other available therapies, and repeated applications of NGX-4010 do not result in an increased incidence of application-site AEs, dermal irritation, intolerability, or impaired neurologic function [32]. Efficacy has been demonstrated when NGX-4010 is used alone as well as in combination with other systemic medications for neuropathic pain. Treatment with NGX-4010 neither increases the pill burden nor the potential for systemic drug–drug interactions, two important considerations for patients with HIV-DSP, who may already be taking several medications. Data from these two phase III clinical trials have led to the approval of NGX-4010 for the treatment of HIV-DSP in the EU and have resulted in NGX-4010 being given a level A efficacy rating in the European Federation of Neurological Sciences guidelines for HIV neuropathy [33].

## Methods

### Enrollment

Details of each of the studies included in this analysis have been reported previously [22,23]. Both studies were approved by institutional review boards/independent ethics committees and conducted in accordance with the ethical principles of the Declaration of Helsinki, Good Clinical Practice guidelines, and applicable regulatory requirements.

Both studies recruited patients who were ≥18 years of age with a diagnosis of HIV-DSP for ≥2 months and an average baseline NPRS score [34] of 3–9. Patients taking other pain medications such as anticonvulsants, non-selective serotonin reuptake inhibitor (non-SSRI) antidepressants (e.g., tricyclic antidepressants, serotonin–norepinephrine reuptake inhibitors), opioids, non-steroidal anti-inflammatory drugs, salicylates, or acetaminophen had to be on stable doses for ≥21 days before patch application and remain on stable doses throughout the study period. Exclusion criteria in both studies included the prior use of NGX-4010 and use of a topical medication on the painful area within 21 days before the NGX-4010 application day.

### Treatment

Patients were randomly assigned to receive treatment with an NGX-4010 (QUTENZA™/Qutenza®) patch (capsaicin 640 μg/cm^2^, 8% w/w; NeurogesX, Inc., San Mateo, CA, USA) or a low-concentration control patch (capsaicin 3.2 μg/cm^2^, 0.04% w/w). The control patch produced local erythema and a burning sensation to provide effective blinding in the studies.

Patients were pre-treated with a topical local anesthetic cream (L.M.X.4 lidocaine 4%; Ferndale Laboratories, Inc., Ferndale, MI, USA) for 60 minutes before patch application. Both the NGX-4010 and control patches were applied for 30, 60, or 90 minutes in study C107 [22] and for 30 or 60 minutes in study C119 [23]. After patch removal, the treatment area was cleansed with a proprietary cleansing gel (NeurogesX, Inc.) formulated to remove residual capsaicin. A rapid-onset, opioid-based oral pain medication (e.g., oxycodone hydrochloride oral solution, 1 mg/ml) could be administered at the onset of treatment-associated discomfort and as needed while in the clinic. Following patch removal, local cooling could be used. Patients could also take a short-term regimen of an opioid-based oral pain medication (e.g., hydrocodone bitartrate/acetaminophen 5 mg/500 mg) for treatment-related discomfort for up to 5 or 7 days post treatment, depending on the study.

### Assessments

The studies included either a ≥5 day [22] or ≥14 day [23] baseline screening period. Assessments were also carried out on the day of treatment (Day 0), at the termination visit (Week 12), and, depending on the study, at interim visits at Weeks 1 and 4 or 4 and 8 during the 12-week blinded observation period. The primary efficacy endpoint was the mean percent change from baseline in NPRS scores for “average pain for the past 24 hours” from Weeks 2–12. Patients recorded their “average pain for the past 24 hours” every evening at 9:00 pm in paper pain diaries from the baseline screening period until the evening before the termination visit.

Other efficacy measurements included the percentage of patients with ≥30% mean decrease in NPRS score from baseline during Weeks 2–12 and the percentage of those feeling improved according to the PGIC at Week 12. In this assessment patients reported how they felt after treatment compared with before treatment on a 7-point scale with −3 indicating “very much worse” to +3 indicating “very much improved” and 0 indicating “no change”.

Safety and tolerability assessments included AEs, vital signs, clinical laboratory tests, physical examination, dermal assessment (0- to 7-point severity score) [24], “pain now” NPRS scores during and after patch application, early patch removal, and use of medication for treatment-related discomfort during Days 0 to 5.

### Efficacy analyses

Data from patients receiving NGX-4010 and control patches for 30 or 60 minutes were integrated from both phase III HIV-DSP studies. The 90-minute NGX-4010 dose was not included in these analyses as it was not evaluated in study C119 [23]. Intent-to-treat efficacy analyses consisted of all patients who received any study treatment and had at least 3 days of available NPRS scores during the baseline period. The primary efficacy endpoint was the percent change in NPRS scores from baseline during Weeks 2–12. Changes in NPRS scores from baseline to Weeks 2–12 were compared between treatment groups using a pre-specified gender-stratified ANCOVA model with baseline pain score, pre-topical anesthetic pain score, and percent change in pain score after topical anesthetic treatment as covariates. To avoid the potentially confounding effect of opioid medications allowed during Days 0–5, Week 1 NPRS scores were not included in the primary endpoint analysis. In addition, least square means of the difference between treatment groups and the 95% confidence interval (CI) were calculated.

Missing post-treatment NPRS scores were imputed using a modified last observation carried forward approach. If the NPRS score was missing on post-treatment Days 0–8 or on Day 8 and one or more consecutive days, then the baseline score was imputed for those days. If the NPRS score was missing for any day after Day 8, then the missing score was imputed by the last available non-imputed score recorded before that day. If all post-treatment NPRS scores were missing (including Day 0), all scores were imputed using the baseline score. NPRS baseline scores were calculated using all available screening scores that were not biased by pain medication changes [22] or using all available screening scores between Day −14 and Day −1 [23]. For the weekly and the daily NPRS scores, missing scores were not imputed.

The percentage of responders (classified as those achieving ≥30% reduction in NPRS score from baseline) was compared between groups using logistic regression analyses, with baseline pain score, gender, pre-topical anesthetic pain score, and percent change in pain score after topical anesthetic treatment as covariates. In addition, odds ratios and the 95% CI of observing responses in the NGX-4010 group compared with the control group were estimated. The percentage of patients reporting improvements according to the PGIC was compared between treatment groups using Fisher’s exact test. For both the primary endpoint and responder analysis, the treatment effect was assessed by gender, baseline pain score (median NPRS score of < and ≥6.07), HIV-DSP duration (median of < and ≥5.2 years), and concomitant neuropathic pain medication use at study entry (defined as receiving an anticonvulsant, non-SSRI antidepressant or opioid on Day −1 and for at least 7 consecutive days) in patients treated for 30 minutes.

### Safety and tolerability analyses

AEs were coded using the Medical Dictionary for Regulatory Activities version 9.0. Medication use for treatment-related discomfort (from Days 0–5) and the percentage of patients completing the intended patch duration were compared using Fisher’s exact test. The percentages of patients reporting each level of dermal response were compared using the Cochran–Mantel–Haenszel test. The maximum changes in “pain now” NPRS score from the pre-topical anesthetic time point during and after patch application were summarized and compared using the Wilcoxon rank sum test.

Patients were analyzed as randomized for the efficacy analyses and as treated for the safety analyses.

## Abbreviations

AE: Adverse event; ANCOVA: Analysis of covariance; CI: Confidence interval; HIV-DSP: HIV-associated neuropathy; LS: Least square; NPRS: Numerical Pain Rating Scale; OR: Odds ratio; PGIC: Patient Global Impression of Change; rhNGF: Recombinant-human nerve growth factor; SD: Standard deviation; SE: Standard error; TRPV1: Transient Receptor Potential Vanilloid 1.

## Competing interests

DMS has received consultancy fees and/or honorarium or speaker fees from NeurogesX, Astellas Pharma Europe Ltd., Eli Lilly and Pfizer. DMS’s institute has received grants from NeurogesX, Astra Zeneca and Pfizer. MF has received speaker fees and funding to attend a conference from Astellas. SB’s institute, AIDS Research Alliance, received grants to conduct the research. GS received funds as site investigator. JKT and GFV are former employees of NeurogesX and still own stock in NeurogesX.

## Authors’ contributions

SB, DMS, GM, BJB, GS, NL, CO, and MF conducted the study, interpreted the data, and edited the manuscript. GFV and JKT designed the study, analyzed and interpreted the data, and prepared the manuscript. All authors read and approved the final manuscript.

## References

[B1] Ferrari S Vento S Monaco S Cavallaro T Cainelli F Rizzuto N Temesgen Z Human immunodeficiency virus-associated peripheral neuropathies Mayo Clin Proc 2006 81 213 219 10.4065/81.2.213 16471077

[B2] Gonzalez-Duarte A Robinson-Papp J Simpson DM Diagnosis and management of HIV-associated neuropathy Neurol Clin 2008 26 821 832 10.1016/j.ncl.2008.04.001 18657728

[B3] McArthur JC Brew BJ Nath A Neurological complications of HIV infection Lancet Neurol 2005 4 543 555 10.1016/S1474-4422(05)70165-4 16109361

[B4] Ellis RJ Rosario D Clifford DB McArthur JC Simpson D Alexander T Gelman BB Vaida F Collier A Marra CM Ances B Atkinson JH Dworkin RH Morgello S Grant I Continued high prevalence and adverse clinical impact of human immunodeficiency virus-associated sensory neuropathy in the era of combination antiretroviral therapy: the CHARTER study Arch Neurol 2010 67 552 558 10.1001/archneurol.2010.76 20457954 PMC3924778

[B5] Kieburtz K Simpson D Yiannoutsos C Max MB Hall CD Ellis RJ Marra CM McKendall R Singer E Dal Pan GJ Clifford DB Tucker T Cohen B A randomized trial of amitriptyline and mexiletine for painful neuropathy in HIV infection. AIDS clinical trial group 242 protocol team Neurology 1998 51 1682 1688 10.1212/WNL.51.6.1682 9855523

[B6] Simpson DM Schifitto G Clifford DB Murphy TK Durso-De Cruz E Glue P Whalen E Emir B Scott GN Freeman R Pregabalin for painful HIV neuropathy: a randomized, double-blind, placebo-controlled trial Neurology 2010 74 413 420 10.1212/WNL.0b013e3181ccc6ef 20124207 PMC2816006

[B7] Estanislao L Carter K McArthur J Olney R Simpson D A randomized controlled trial of 5% lidocaine gel for HIV-associated distal symmetric polyneuropathy J Acquir Immune Defic Syndr 2004 37 1584 1586 10.1097/00126334-200412150-00010 15577414

[B8] Paice JA Ferrans CE Lashley FR Shott S Vizgirda V Pitrak D Topical capsaicin in the management of HIV-associated peripheral neuropathy J Pain Symptom Manage 2000 19 45 52 10.1016/S0885-3924(99)00139-6 10687326

[B9] Abrams DI Jay CA Shade SB Vizoso H Reda H Press S Kelly ME Rowbotham MC Petersen KL Cannabis in painful HIV-associated sensory neuropathy: a randomized placebo-controlled trial Neurology 2007 68 515 521 10.1212/01.wnl.0000253187.66183.9c 17296917

[B10] Ellis RJ Toperoff W Vaida F van den BG Gonzales J Gouaux B Bentley H Atkinson JH Smoked medicinal cannabis for neuropathic pain in HIV: a randomized, crossover clinical trial Neuropsychopharmacology 2009 34 672 680 10.1038/npp.2008.120 18688212 PMC3066045

[B11] Ware MA Wang T Shapiro S Robinson A Ducruet T Huynh T Gamsa A Bennett GJ Collet JP Smoked cannabis for chronic neuropathic pain: a randomized controlled trial CMAJ 2010 182 E694 E701 10.1503/cmaj.091414 20805210 PMC2950205

[B12] O'Connor AB Dworkin RH Treatment of neuropathic pain: an overview of recent guidelines Am J Med 2009 122 10 Suppl S22 S32 19801049 10.1016/j.amjmed.2009.04.007

[B13] Koeppe J Armon C Lyda K Nielsen C Johnson S Ongoing pain despite aggressive opioid pain management among persons with HIV Clin J Pain 2010 26 190 198 10.1097/AJP.0b013e3181b91624 20173432

[B14] Phillips TJ Cherry CL Cox S Marshall SJ Rice AS Pharmacological treatment of painful HIV-associated sensory neuropathy: a systematic review and meta-analysis of randomised controlled trials PLoS One 2010 5 e14433 10.1371/journal.pone.0014433 21203440 PMC3010990

[B15] Schifitto G Yiannoutsos C Simpson DM Adornato BT Singer EJ Hollander H Marra CM Rubin M Cohen BA Tucker T Koralnik IJ Katzenstein D Haidich B Smith ME Shriver S Millar L Clifford DB McArthur JC Long-term treatment with recombinant nerve growth factor for HIV-associated sensory neuropathy Neurology 2001 57 1313 1316 10.1212/WNL.57.7.1313 11591856

[B16] Caterina MJ Julius D The vanilloid receptor: a molecular gateway to the pain pathway Annu Rev Neurosci 2001 24 487 517 10.1146/annurev.neuro.24.1.487 11283319

[B17] Polydefkis M Yiannoutsos CT Cohen BA Hollander H Schifitto G Clifford DB Simpson DM Katzenstein D Shriver S Hauer P Brown A Haidich AB Moo L McArthur JC Reduced intraepidermal nerve fiber density in HIV-associated sensory neuropathy Neurology 2002 58 115 119 10.1212/WNL.58.1.115 11781415

[B18] Ma W Zhang Y Bantel C Eisenach JC Medium and large injured dorsal root ganglion cells increase TRPV-1, accompanied by increased alpha2C-adrenoceptor co-expression and functional inhibition by clonidine Pain 2005 113 386 394 10.1016/j.pain.2004.11.018 15661448

[B19] Bley KR Gomtsyan A, Faltynek CR TRPV1 agonist approaches for pain management Vanilloid Receptor TRPV1 in Drug Discovery 2010 Hoboken, NJ, USA: John Wiley & Sons, Inc 325 347

[B20] Szallasi A Blumberg PM Vanilloid (capsaicin) receptors and mechanisms Pharmacol Rev 1999 51 159 212 10353985

[B21] Kennedy WR Vanhove GF Lu SP Tobias J Bley KR Walk D Wendelschafer-Crabb G Simone DA Selim MM A randomized, controlled, open-label study of the long-term effects of NGX-4010, a high-concentration capsaicin patch, on epidermal nerve fiber density and sensory function in healthy volunteers J Pain 2010 11 579 587 10.1016/j.jpain.2009.09.019 20400377

[B22] Simpson DM Brown S Tobias J Controlled trial of high-concentration capsaicin patch for treatment of painful HIV neuropathy Neurology 2008 70 2305 2313 10.1212/01.wnl.0000314647.35825.9c 18541884

[B23] Clifford DB Simpson DM Brown S Moyle G Brew BJ Conway B Tobias JK Vanhove GF A randomized, double-blind, controlled study of NGX-4010, a capsaicin 8% dermal patch, for the treatment of painful HIV-associated distal sensory polyneuropathy J Acquir Immune Defic Syndr 2012 59 126 133 10.1097/QAI.0b013e31823e31f7 22067661

[B24] US Food and Drug Administration Center for Drug Evaluation and Research Guidance for industry: Skin irritation and sensitization testing of generic transdermal drug products 1999 Washington, DC, USA: US Department of Health and Human Services http://www.fda.gov/ohrms/dockets/98fr/990236Gd.pdf#search=%22HillTop%20Research%2C%20Inc.%20dermal%20irritation%22

[B25] Dworkin RH Turk DC Farrar JT Haythornthwaite JA Jensen MP Katz NP Kerns RD Stucki G Allen RR Bellamy N Carr DB Chandler J Cowan P Dionne R Galer BS Hertz S Jadad AR Kramer LD Manning DC Martin S McCormick CG McDermott MP McGrath P Quessy S Rappaport BA Robbins W Robinson JP Rothman M Royal MA Simon L Stauffer JW Stein W Tollett J Wernicke J Witter J Core outcome measures for chronic pain clinical trials: IMMPACT recommendations Pain 2005 113 9 19 10.1016/j.pain.2004.09.012 15621359

[B26] Haanpää M Attal N Backonja M Baron R Bennett M Bouhassira D Cruccu G Hansson P Haythornthwaite JA Iannetti GD Jensen TS Kauppila T Nurmikko TJ Rice AS Rowbotham M Serra J Sommer C Smith BH Treede RD NeuPSIG guidelines on neuropathic pain assessment Pain 2011 152 14 27 10.1016/j.pain.2010.07.031 20851519

[B27] Backonja M Wallace MS Blonsky ER Cutler BJ Malan P Jr Rauck R Tobias J NGX-4010, a high-concentration capsaicin patch, for the treatment of postherpetic neuralgia: a randomised, double-blind study Lancet Neurol 2008 7 1106 1112 10.1016/S1474-4422(08)70228-X 18977178

[B28] Webster LR Malan TP Tuchman MM Mollen MD Tobias JK Vanhove GF A multicenter, randomized, double-blind, controlled dose finding study of NGX-4010, a high-concentration capsaicin patch, for the treatment of postherpetic neuralgia J Pain 2010 11 972 982 20655809 10.1016/j.jpain.2010.01.270

[B29] Gilron I Bailey JM Tu D Holden RR Weaver DF Houlden RL Morphine, gabapentin, or their combination for neuropathic pain N Engl J Med 2005 352 1324 1334 10.1056/NEJMoa042580 15800228

[B30] Gilron I Bailey JM Tu D Holden RR Jackson AC Houlden RL Nortriptyline and gabapentin, alone and in combination for neuropathic pain: a double-blind, randomised controlled crossover trial Lancet 2009 374 1252 1261 10.1016/S0140-6736(09)61081-3 19796802

[B31] Vanhove GF Wallace M Irving G Backonja M Webster LR Tobias JK Integrated safety analyses of NGX-4010, an 8% capsaicin patch, in patients with peripheral neuropathic pain13th World Congress on Pain; August 29-September 2, 2010 2010 Montréal, Québec, Canada Abstract [PH 101]

[B32] Simpson DM Gazda S Brown S Webster LR Lu SP Tobias JK Vanhove GF Long-term safety of NGX-4010, a high-concentration capsaicin patch, in patients with peripheral neuropathic pain J Pain Symptom Manage 2010 39 1053 1064 10.1016/j.jpainsymman.2009.11.316 20538187

[B33] Attal N Cruccu G Baron R Haanpaa M Hansson P Jensen TS Nurmikko T EFNS guidelines on the pharmacological treatment of neuropathic pain: 2010 revision Eur J Neurol 2010 17 1113 1188 10.1111/j.1468-1331.2010.02999.x 20402746

[B34] Farrar JT Young JP Jr LaMoreaux L Werth JL Poole RM Clinical importance of changes in chronic pain intensity measured on an 11-point numerical pain rating scale Pain 2001 94 149 158 10.1016/S0304-3959(01)00349-9 11690728

